# Anticancer potential of *Ulva lactuca* extract: inhibition of growth kinetics and radial invasion in 3D tumor spheroids

**DOI:** 10.1007/s44164-026-00120-2

**Published:** 2026-04-22

**Authors:** You-shin Chen, Dan Xun, Shupeng Zhang, Rithvik Venisetty, Naveen Vattipally, Heather Dawson, Dennis Viele

**Affiliations:** 1 Department of Biology, University of Michigan-Flint, Flint, 48502 USA; 2 Department of Biochemistry and Molecular & Cellular Biology, Georgetown University, Washington, DC 20057 USA

**Keywords:** Anticancer, *Ulva lactuca*, 3D tumor spheroids, Tumor proliferation, Growth kinetics, Radial invasion, Marine natural products

## Abstract

**Purpose:**

Cancer is a leading cause of global mortality, characterized by uncontrolled proliferation and metastasis. Novel therapeutics are necessary to combat this heterogeneous disease. Traditional pre-clinical studies often utilize two-dimensional (2D) cell culture models that do not mimic the complex architecture of human tumors. This study aimed to investigate the anticancer potential of an aqueous extract from the green seaweed *Ulva lactuca*, specifically focusing on its ability to modulate growth kinetics and restrict radial invasion in a more physiologically relevant three-dimensional (3D) environment.

**Methods:**

We employed a 3D multicellular tumor spheroid model across three human malignancy lines: HT-29 (colorectal adenocarcinoma), LNCaP (lymph node carcinoma of the prostate), and HT-1080 (fibrosarcoma). Spheroids were treated with and without *U. lactuca* extract and monitored longitudinally for 14 days. Growth kinetics were characterized using locally estimated scatterplot smoothing (LOESS) regression and area-under-the-curve (AUC) analysis. In addition, a 3D invasion assay was performed using HT-1080 spheroids to quantify the extract’s impact on radial expansion and volumetric infiltration.

**Results:**

*U. lactuca* extract significantly inhibited spheroid expansion across all cell lines, with growth reductions ranging from 20% to 26% (*p <* 0.001). Longitudinal analysis revealed substantial kinetic delays, with doubling time milestones extended by up to 3.5 days in HT-29 models. In the HT-1080 invasion assay, the extract suppressed invasive area by 42.5% (*p <* 0.001). Notably, treatment delayed the radial expansion milestone by four days, effectively reducing the invasion burden compared to untreated controls.

**Conclusion:**

These findings demonstrate that *U. lactuca* extract inhibits the proliferative and invasive trajectories of multiple cancer types in 3D models. By significantly altering growth kinetics and restricting radial invasion, this marine-derived extract shows promise as a multi-targeted therapeutic agent against aggressive malignancies.

## Introduction

Every minute an estimated 38 people are diagnosed with cancer globally, according to the International Agency for Research on Cancer (IARC) of the World Health Organization (WHO) in 2022 [1]. With the 5-year net survival rate for 11 major cancers remaining at approximately 42.6% as of 2020, there is a critical need for additional treatment options [2]. Marine natural products have emerged as a valuable source of therapeutic agents, leading to the clinical approval of anti-cancer drugs, including Cytarabine for acute leukemia [3, 4], Trabectedin for soft tissue sarcoma [5] and ovarian cancer [6], as well as Eribulin mesylate for metastatic breast cancer [7] and liposarcoma [8].

This study aims to investigate the growth inhibitory and anti-invasion effects of water-soluble compounds from *Ulva lactuca*, a green macroalgae commonly known as sea lettuce. The physiological resilience of *Ulva*—characterized by high UV tolerance and rapid biomass accumulation—positions it as a robust and sustainable bio-resource for drug discovery. While the alcoholic fraction of *Ulva* contains lipophilic antioxidants such as carotenoids and certain flavonoids, this study focuses on the water-soluble fraction, primarily dominated by the sulfated heteropolysaccharide ulvan. The building blocks of ulvan include sulfated L-rhamnose, D-glucuronic acid, L-iduronic acid, D-xylose and sulfate [9]. Given ulvan’s high concentration (up to 9 to 36% dry weight) [10, 11], and its structural similarity to mammalian glycosaminoglycans, it represents a high-priority target for aqueous-based therapeutic extraction.

Studies have shown that macroalgae of the genus *Ulva* can inhibit growth in various cancer cell lines through different mechanisms. *U. rigida* polysaccharides reduced the viability of cells from colorectal carcinoma (HCT 116; IC_50_ = 0.1 $\:\pm\:$ 0.02 mg/mL), myeloid leukemia (U-937; IC_50_ = 2.4 $\:\pm\:$ 0.4 mg/mL), and malignant melanoma (G-361; IC_50_ = 4.3 $\:\pm\:$ 1.2 mg/mL) [12]. Polysaccharides isolated from *U. lactuca* has been shown to reduce cell viability of hepatocarcinoma (HepG2; IC_50_ = 55.56 µg/mL and 29.67 $\:\pm\:$ 2.87 µg/mL) [13, 14], colorectal carcinoma (HCT 116; IC_50_ = 22.65 µg/mL) [13], cervical adenocarcinoma (HeLa; IC_50_ = 36.33 $\:\pm\:$ 3.83 µg/mL) [14], MCF-7 (IC_50_ = 25.09 $\:\pm\:$ 1.36 µg/mL and 224.716 µg/mL) [14, 15], and MDA-MB-231 (IC_50_ *>* 1000 µg/mL [16], in a dose-dependent manner. Treating with ulvan isolated from *U. lactuca* (500 µg/mL) before cells adhered to a tissue culture plate reduced the number of colonies formed by colorectal adenocarcinoma cells (HT-29) [17]. However, in the same study, *U. lactuca* extract exhibited a bi-phasic effect, in which low concentrations (1–500 µg/mL) of ulvan stimulated HT-29 cell proliferation and high concentration (5,000 µg/mL) of ulvan reduced cell proliferation, using a neutral red viability assay [17].

In vivo,* U. lactuca* was shown to induce the expression of apoptotic marker, p53, and suppress the expression of the anti-apoptotic marker, Bcl2, in the 7,12-dimethylbenz[a]anthracene (DMBA)-induced breast cancer model in Wistar rats [15]. In a H22 hepatoma mouse xenograft model, *U. lactuca* polysaccharide treatment has been shown to reduce tumor growth by altering gut microbiome population and metabolism [18]. Proteomics and immunohistochemistry studies on these xenograft tumors revealed induced expression of CD5L and activation of complement immunity [19]. Collectively, these findings underscore the potential of *Ulva*-derived bioactives as potent anti-neoplastic agents.

While algal bioactive compounds have demonstrated significant anti-cancer potentials [20–23], to our best knowledge, this is the first study to utilize extracellular matrix(ECM)-embedded 3-dimensional (3D) tumor spheroid models to evaluate the anti-cancer efficacy of *U. lactuca* aqueous extract. A previous investigation utilized HT-29 spheroids in a rotating bioreactor; however, that model relied on Cytodex-3 microcarriers—gelatin-coated dextran beads that serve as a rigid synthetic core. This ‘hollow-sphere’ geometry limits cellular growth to an outward-facing monolayer, preventing the formation of a solid, scaffold-free tumor mass and the inner necrotic core characteristic of in vivo tumors [17].

In preclinical studies, tumor microenvironment is better simulated using 3D cell culture models, compared to 2D cell culture models, due to differences in spatial architecture and physiochemical gradients. Interactions between tumor cells and ECM present in 3D models trigger signaling pathways that regulate growth and survival, which are absent in 2D models [24, 25]. The dynamic network of macromolecules in ECM, including glycoproteins, proteoglycans, glycosaminoglycans, and integrins, not only provides structural scaffolding for tumor cells, but also actively mediates a complex array of signaling cues between tumor microenvironment and tumor cells to regulate tumor cell behavior, including the transition from a proliferative to a migratory phenotype.

Unlike the uniform environment of 2D cultures, 3D spheroids establish physiologically relevant gradients that mirror the complexity of solid tumors. Oxygen and nutrients must diffuse inward, leading to a stratified architecture where a highly proliferative outer shell surrounds a quiescent middle layer and a necrotic core. Conversely, metabolic waste products such as lactic acid accumulate at the center, lowering the local pH and activating hypoxia-inducible factors. These factors trigger the expression of efflux pumps, which can alter drug efficacy. Consequently, therapeutic efficacy is challenged not only by physical barriers but also biological defenses [26]. Agents must penetrate a dense ECM and navigate tight cell-cell junctions—obstacles that are absent in the uniform exposure provided by 2D models.

Furthermore, research demonstrates that gene expression profiles in 3D models more closely resemble patient biopsies compared to traditional 2D cultures, including DNA methylation patterns and microRNA expression shown in colorectal cancer [27], and key glioma markers like *GFAP*, *CD44*, and *PTEN* in diffuse glioma [28]. Additionally, differentially expressed gene (DEG) analysis using Spearman correlation coefficients demonstrates that adenocarcinoma cells cultured as 3D spheroids share a significantly higher transcriptomic association with primary human lung adenocarcinoma specimens than their 2D monolayer counterparts [29].

Despite the clear physiological advantages of 3D modeling, a significant knowledge gap persists in marine pharmacognosy. While numerous studies have utilized traditional 2D monolayers to establish the anti-proliferative effects of *U*. *lactuca* on various cancer cell lines, we utilize 3D tumor spheroids to evaluate the anti-cancer efficacy of *U. lactuca* extract. This distinction is critical because 3D spheroids are characterized by increased chemoresistance compared to 2D cultures, largely due to the limited diffusion of therapeutic agents through the dense extracellular matrix and the presence of quiescent cells in the hypoxic core [30]. Demonstrating efficacy in a 3D context, therefore, provides a much more rigorous and clinically relevant validation of the extract’s potency.

This study addresses three pathologies with unmet clinical needs: fibrosarcoma (HT-1080), colorectal adenocarcinoma (HT-29), and prostate adenocarcinoma (LNCaP). Fibrosarcoma remains difficult to treat due to its aggressive local infiltration and high recurrence rates [31]. Colorectal cancers present a major challenge through ‘multidrug resistance’, as solid tumor architecture shields cells from chemotherapy. Furthermore, prostate cancer requires new options to disrupt self-sustaining, hormone-modulated growth driven by intrinsic signaling. By evaluating these specific challenges—invasion, architectural resistance, and growth in complex environments—this study provides a more comprehensive assessment of *U. lactuca* aqueous extract. This approach offers a preliminary exploration of its anti-neoplastic potential within models that more closely reflect the physiological hurdles of solid tumors.

In this study, we hypothesized that the water-soluble extract of *U. lactuca* would significantly inhibit growth and invasion in 3D models of fibrosarcoma, colorectal, and prostate cancers. We anticipated that the bioactive compounds, especially sulfated polysaccharides in aqueous algal extract would disrupt established growth kinetics—manifesting as extended doubling times and reduced cumulative tumor burden—while simultaneously arresting the radial infiltration of cells into the surrounding matrix, thereby demonstrating the therapeutic potential of marine-derived compounds in a physiologically relevant tumor microenvironment.

## Materials and methods

### Cell culture

Colorectal adenocarcinoma (HT-29), lymph node carcinoma of the prostate (LNCaP) and fibrosarcoma (HT-1080) cell lines were purchased from Lambadi Center Tissue Share Resource, Georgetown University. HT-29, LNCaP, and HT-1080 were then cultured on 100 mm plates (CORNING, Cat. No. 353003) in a 37 °C and 5% CO_2_ cell culture incubator, using McCoy’s 5 A (Hyclone, Cat. No.SH30200.01), RPMI (Hyclone, Cat. No. SH300270.02) and EMEM (CORNING, Cat. No.10-009-CV) medium, respectively. All media were supplemented with heat-inactivated 10% FBS (Sigma, Cat. No. F2442, Lot No.130349) and 1% Penicillin/Streptomycin (CORNING, Cat. No. 30-001-CI). When cells reached 80% confluence, cells were sub-cultured. Cells were sub-cultured every 2–3 days with a 1 to 4 split each time. DPBS (CORNING, Cat. No. 21–031-CV) was used to wash cells followed by Trypsin-EDTA (CORNING, Cat. No. 25–053-CI) protease activity that can make the adherent cells detach from the plate.

### Aqueous algal extract production

*Ulva lactuca* was purchased from UTEX Culture Collection of Algae (CCA) and cultured in a photobioreactor with a 16 h:8 h LED light: dark photoperiod (Grofizz, UTEX CCA) using Erdschreiber’s Medium (UTEX). Algae growth was traced through measuring chlorophyll amount at A430 and A670. Specifically, 10 mL of algae culture was initially centrifuged at 8,000 rpm for 5 min to pellet the algae and washed three times with deionized (DI) water. The washed algae pellet was resuspended in methanol and centrifuged to obtain supernatant. The absorbance values at A430 and A670 were then measured. *U. lactuca* was harvested at the early stationary phase. 500 mL of *U. lactuca* culture was then centrifuged at 4000 rpm for 10 min and the cell pellet was dried down at 40 °C overnight. One gram of dry *U. lactuca* was added to 16 mL of distilled water (1:16 w/v ratio; 62.5 mg/mL) and autoclaved for 1 h at 121 °C. The 1:16 w/v ratio was chosen to be close to the published protocol [10]. Afterwards, aqueous algal extract was separated from the insoluble components by centrifugation at 4000 rpm for 5 min. The extract was stored at 4 °C for future use.

### Spheroid growth curve

Two thousand of each line of cancer cells were embedded in Spheroid Formation Matrix (R&D SYSTEMS, 3500-096-K) and plated onto a 96-well Ultra Low Attachment (ULA) plate with round bottom wells (CORNING, 7007). After cells assembled into a compact spheroid on Day 3 or Day 4, each spheroid was incubated with either cell culture medium containing crude algae extract (10% v/v; 6.25 mg/mL) or sterile DI water (10% v/v). Since ulvan is 9–36% dry *U. lactuca*, the ulvan concentration in our crude extract was 0.56–2.25 mg/mL [10]. A single, fixed concentration was selected to establish proof-of-concept efficacy in a diffusion-limited 3D system rather than to define a precise IC₅₀, which will be addressed in future dose–response studies. Spheroids were imaged under a microscope for 10–11 days after treatment. Overall spheroid size was measured using ImageJ software to quantify the area occupied by cells.

### Spheroid invasion assay

Two thousand HT-1080 cells were embedded in Spheroid Formation Matrix (R&D SYSTEMS, 3500-096-K) and plated onto a 96-well ULA plate with round bottom wells (CORNING, 7007). After cells assembled into a compact spheroid on Day 4, Invasion Matrix (R&D SYSTEMS, 3500-096-K) was added to each well, following instruction manual. Each spheroid was then incubated with either cell culture medium containing crude algae extract (10% v/v; 6.25 mg/mL) or sterile DI water (10% v/v). Since ulvan is 9–36% dry *U. lactuca*, the ulvan concentration in our crude extract was 0.56–2.25 mg/mL [10]. A single, fixed concentration was selected to establish proof-of-concept inhibition of ECM-driven invasion in a diffusion-limited 3D spheroid model, rather than to define a precise IC₅₀, which will be addressed in future studies. Spheroids were imaged under a microscope for 10 days after treatment. Overall spheroid size was measured using ImageJ software to quantify the area occupied by cells.

### Statistical analyses

The following analysis evaluates the anti-proliferative efficacy of *U. lactuca* algal extract across three distinct human malignancies: fibrosarcoma (HT-1080), colorectal adenocarcinoma (HT-29), and lymph node carcinoma of the prostate (LNCaP). Linear mixed-effects models accounting for repeated measures were used to investigate if treatment with aqueous algae extract has a statistically significant decrease in tumor spheroid size and invasion area. Moreover, to provide a comprehensive assessment of therapeutic impact, we utilized locally estimated scatterplot smoothing (LOESS) to characterize not only absolute changes in spheroid size but also the kinetics of growth, including doubling time delays and cumulative tumor burden. The evaluation was also on the invasiveness of HT-1080 cells in a 3D invasion assay using LOESS to compare the total invaded area and invasive burden before and after *U. lactuca* aqueous extract treatment. By integrating visual analysis of 95% confidence intervals with longitudinal growth data, this section establishes the statistical robustness and clinical relevance of the extract’s inhibitory effects across varied cancer pathologies. Each experiment was performed at least twice with 3 to 5 biological replicates each time. *P*-values lower than 0.05 are considered statistically significant.

## Results

### Aqueous extract of *U. lactuca* reduced tumor spheroid size in human fibrosarcoma cell line HT-1080

**Fig. 1:**
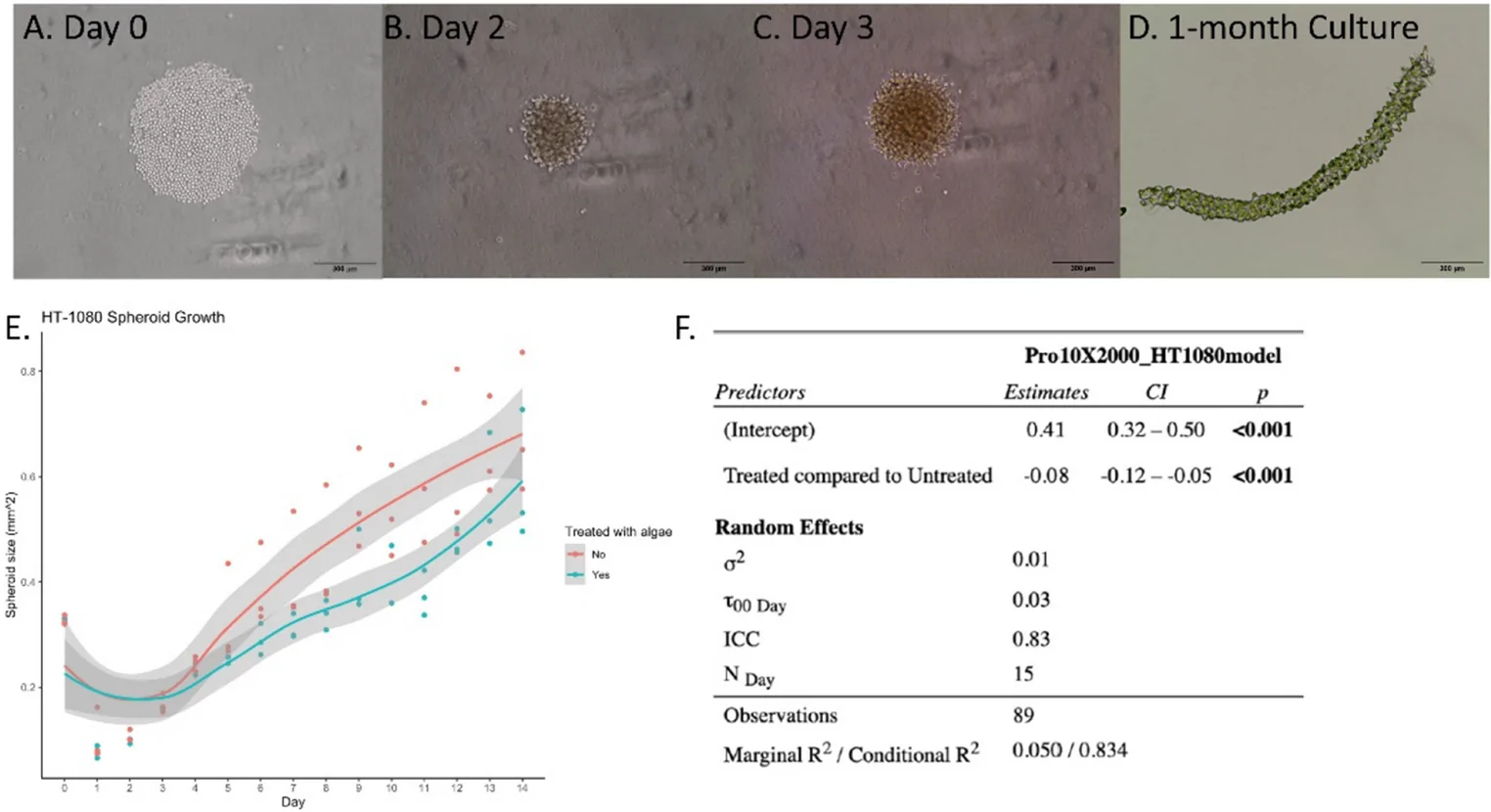
*Ulva Lactuca* Extract Treatment Reduced HT-1080 Tumor Spheroid Size. On Day 0, 2,000 HT-1080 cells were aggregated by centrifugation (**A**). Each spheroid was photographed under a microscope and spheroid area was monitored daily for 2 weeks (n = 3 for each group). The compacted spheroid increased in size between Day 2 and Day 3 (**B–C**). *U. lactuca* algae culture was established successfully in our laboratory and used to produce aqueous algal extract. Algae picture here was taken after culturing for 1 month. (**D**). On Day 3, HT-1080 fibrosarcoma spheroids were treated with and without aqueous *U. lactuca* algal extract. Solid lines represent the best-fit line for spheroid size over time calculated using LOESS. The gray shaded regions indicate the 95% confidence intervals (**E**). Statistical analysis using a linear mixed-effects model confirmed a significant reduction in the spheroid size of the treated group compared to the control (p < 0.001; F). The model estimated a significant fixed effect of treatment (−0.08mm^2^; 95% CI: −0.12 to −0.05), indicating a consistent reduction in tumor burden between Day 3 and Day 14

Each fibrosarcoma HT-1080 spheroid was established from 2,000 cells per well in a ULA well in a 96-well plate (Fig. 1 A). The surface area occupied by HT-1080 cells was measured daily for 15 days. Following an initial compaction phase between Day 0 and Day 1, the scatterplot showed a period of spheroid expansion through Day 3 (Fig. 1B*–*C, E). On day 3, HT-1080 tumor spheroids were treated with or without *U. lactuca* algal extract. Analysis using LOESS revealed a progressive divergence in spheroid size between the two groups from Day 4 to Day 11 (Fig. 1E), indicating a sustained and compounding treatment effect. Overall, the spheroid growth was reduced by 20% in the treated group, compared to the untreated group (0.4 mm^2^ vs. 0.5 mm^2^) between Day 3 and Day 14. The model revealed a statistically significant reduction in spheroid size after *U. lactuca* extract treatment (*p <* 0.001; Fig. 1F). The statistical robustness of this inhibition is further evidenced by the 95% confidence intervals (gray shaded areas), which remained entirely non-overlapping between Day 7 and Day 12.

In addition to the absolute size reduction, the LOESS curves revealed a significant spheroid progression delay in the treated group. While both groups reached a baseline size of 0.18 mm² on Day 3, the treated spheroids exhibited a two-day delay in doubling their size (Day 8.5) compared to the control (Day 6.5). This slower division rate limits spheroid expansion and resulted in a significantly reduced Area Under the Curve (AUC). By suppressing key growth milestones, the *U. lactuca* treatment lowered cumulative tumor burden throughout the study, reflecting sustained inhibition of tumor growth over the experimental timeframe. 

### Aqueous extract of *U. lactuca* reduced tumor spheroid size in human colorectal adenocarcinoma cell line HT-29

**Fig. 2:**
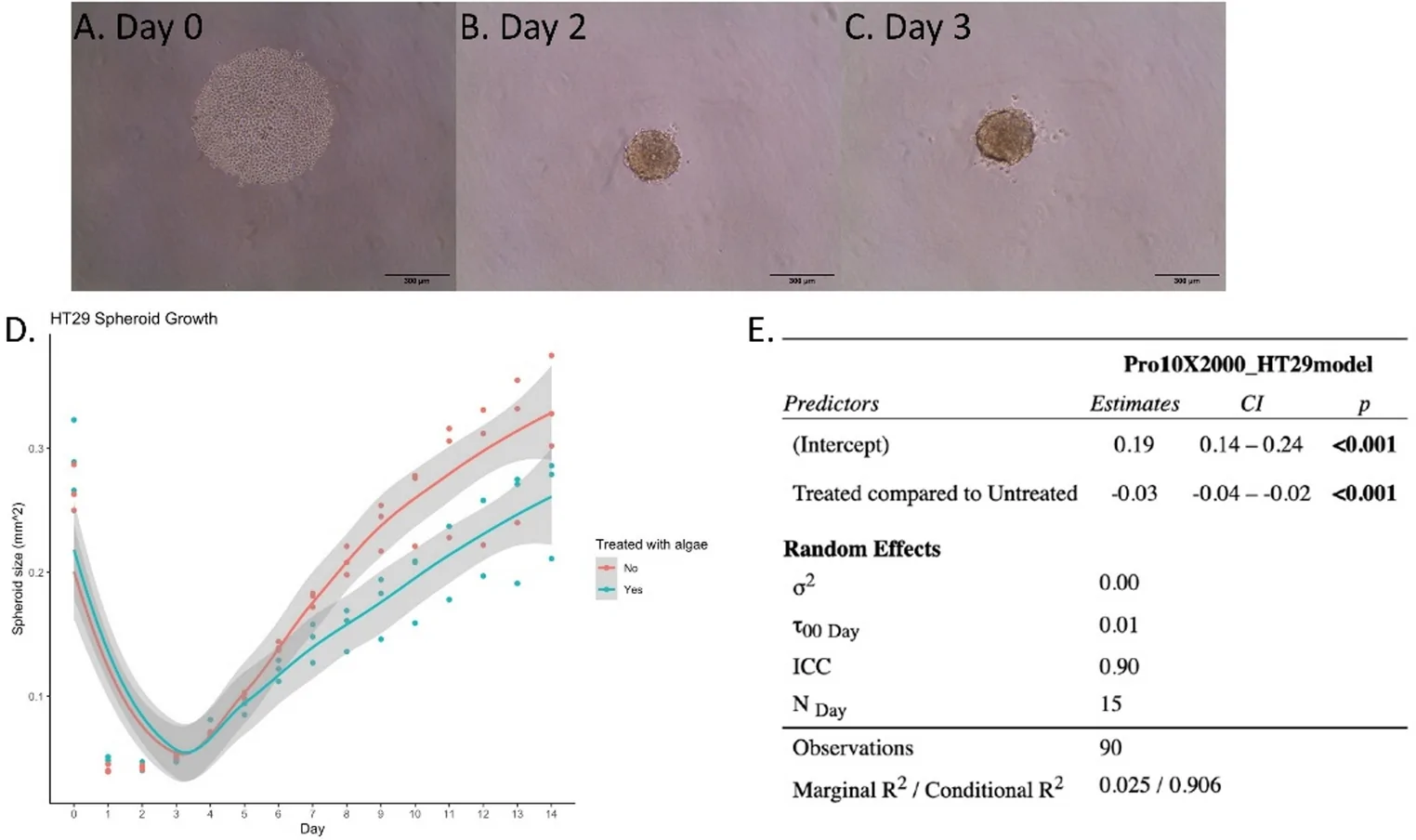
*Ulva Lactuca* Extract Treatment Reduced HT-29 Tumor Spheroid Size. On Day 0, 2,000 HT-29 cells were aggregated by centrifugation (**A**). Each spheroid was photographed under a microscope and spheroid area was monitored daily for 2 weeks (*n* = 3 for each group). The compacted spheroid increased in size over time between Day 2 and Day 3 (**B**–**C**). On Day 4, HT-29 spheroids were treated with and without aqueous *U. lactuca* algal extract. Solid lines represent the best-fit line for spheroid size over time calculated using LOESS. The gray shaded regions indicate the 95% confidence intervals (**D**). Statistical analysis using a linear mixed-effects model confirmed a significant reduction in the spheroid size of the treated group compared to the control (*p* < 0.001; E). The model estimated a significant fixed effect of treatment (−0.03 mm^2^; 95% CI: −0.04 to −0.02), indicating a consistent reduction in tumor burden between Day 4 to Day 14

The colorectal HT-29 spheroid model was utilized to evaluate the anti-neoplastic potential of the *U. lactuca extract* over a 15-day experiment (2000 cells/well; Fig. 2A). Following a standardized stabilization period (Fig. 2B–C), treatment was applied on Day 4. Analysis using LOESS revealed a progressive divergence in spheroid size starting from Day 4, with the most pronounced separation between Day 7 and Day 13 (Fig. 2D). *U. lactuca* extract treatment reduced HT-29 spheroid size by 26% overall, compared to the untreated group on between Day 3 and Day 14 (0.2 mm^2^ vs. 0.27 mm^2^). LOESS model revealed a statistically significant reduction in spheroid size after *U. lactuca* extract treatment (*p <* 0.001; Fig. 2E). 95% confidence intervals (shaded gray regions) remained entirely isolated from one another from Day 8 through the final observation. The progressive widening of the gap between the intervals further illustrates the sustained and compounding efficacy of the *U. lactuca* extract throughout the exponential growth phase.

Growth suppression was reflected by progressively increasing delays in spheroid expansion over time. While the initial doubling from baseline (0.06 to 0.12 mm^2^) showed a modest 0.5-day delay following *U. lactuca* extract treatment, the subsequent doubling to 0.24 mm^2^ was delayed by 3.5 days, compared to untreated controls (Day 12.5 vs. Day 9). This pronounced proliferative delay in proliferative progression limits spheroid expansion and resulted in a significant reduction in the integrated tumor burden, as measured by AUC. By suppressing successive growth milestones, *U. lactuca* treatment maintained a lower cumulative spheroid mass throughout the experimental timeframe.

### Aqueous extract of *U. lactuca* reduced tumor spheroid size in human prostate carcinoma cell line LNCaP

**Fig. 3:**
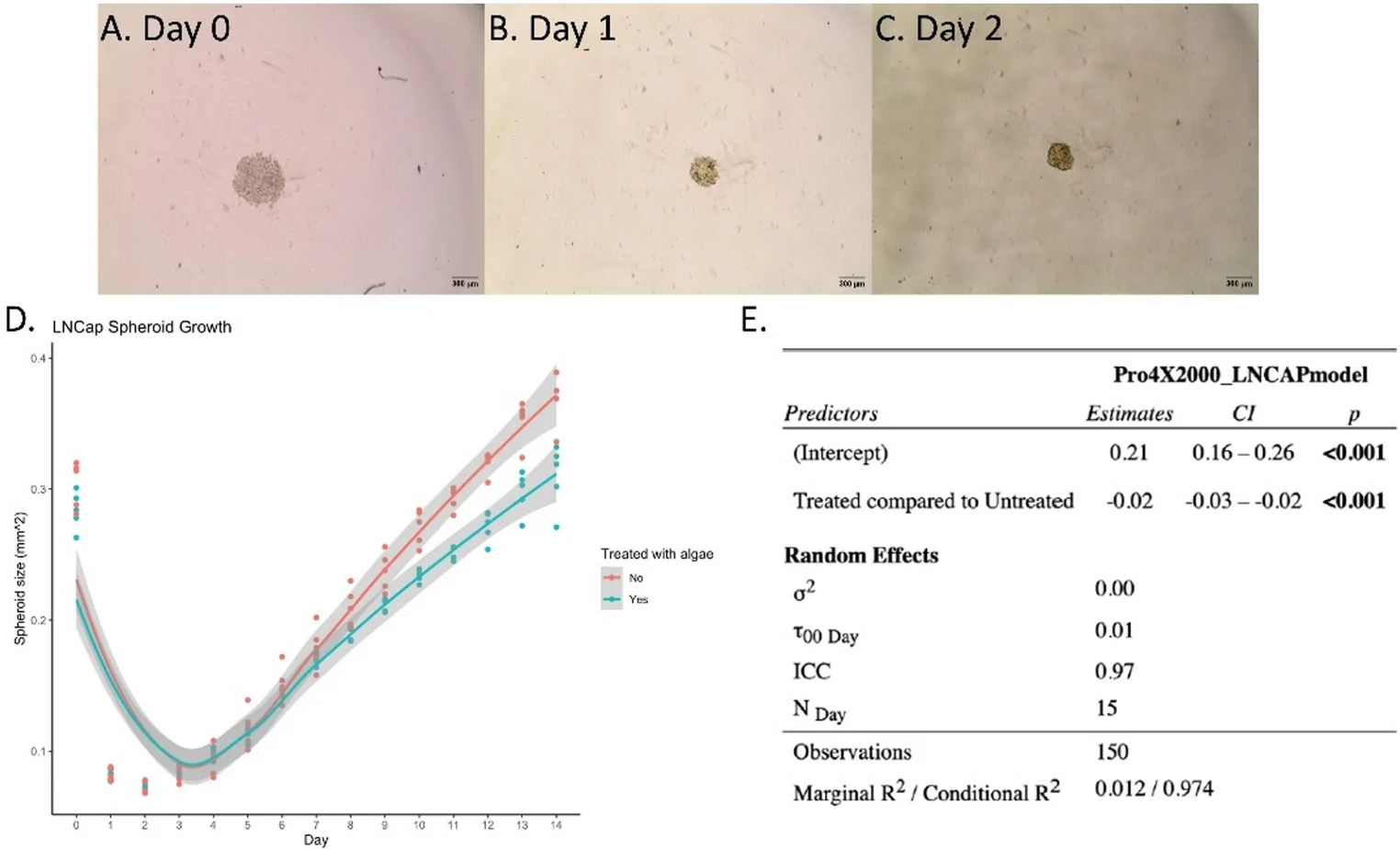
*Ulva Lactuca* Extract Treatment Reduced LNCaP Tumor Spheroid Size. On Day 0, 2,000 LNCaP cells were aggregated by centrifugation (**A**). Each spheroid was photographed under a microscope and spheroid area was monitored daily for 2 weeks (*n* = 3 for each group). The compacted spheroid increased in size over time between Day 1 and Day 2 (**B**–**C**). On Day 3, LNCaP spheroids were treated with and without aqueous *U. lactuca* algal extract. Solid lines represent the best-fit line for spheroid size over time calculated using LOESS. The gray shaded regions indicate the 95% confidence intervals (**D**). Statistical analysis using a linear mixed-effects model confirmed a significant reduction in the spheroid size of the treated group compared to the control (*p* < 0.001; E). The model estimated a significant fixed effect of treatment (−0.02 mm^2^; 95% CI: −0.03 to −0.02), indicating a consistent reduction in tumor burden between Day 3 to Day 14

For the LNCaP prostate adenocarcinoma model, spheroids were established using 2,000 cells per well (Fig. 3A) and monitored for 15 days to track morphological changes. Following the initial compaction phase (Fig. 3B–C), *U. lactuca* treatment was initiated on Day 3. The resulting LOESS analysis demonstrated a pronounced separation in growth trajectories beginning on Day 7, with the most pronounced divergence between Day 9 and Day 14 (Fig. 3D). While the untreated group entered a rapid expansion phase, the treated spheroids showed a flattened growth curve, resulting in a overall 21% smaller surface area between Day 3 and Day 14 (0.22 mm^2^ vs. 0.28 mm^2^). This inhibitory effect was statistically significant, as evidenced by a *p*-value < 0.001 (Fig. 3E). Furthermore, the 95% confidence intervals (gray shaded bands) showed a definitive separation from Day 9 through Day 14; the lack of overlap during this period confirms that the extract’s impact was consistent and statistically distinct from the natural variability of the control group.

This suppression is further highlighted by the disparity of growth milestones. While the initial doubling from baseline (0.09 mm^2^ to 0.18 mm^2^) showed a modest 0.5-day delay, a marked divergence emerged during the subsequent growth phase. Untreated spheroids achieved a second doubling (0.36 mm^2^) by Day 14, whereas the treated group remained substantially growth-restricted, expanding only 1.7-fold from baseline and failing to reach the second doubling threshold before the study’s conclusion. By preventing the attainment of this later growth milestone, *U. lactuca* treatment significantly reduced the cumulative tumor burden, as quantified by AUC, reflecting sustained suppression of spheroid expansion over the course of the study.

### Aqueous extract of *U. lactuca* reduced tumor spheroid invasiveness in human fibrosarcoma cell line HT-1080

**Fig. 4:**
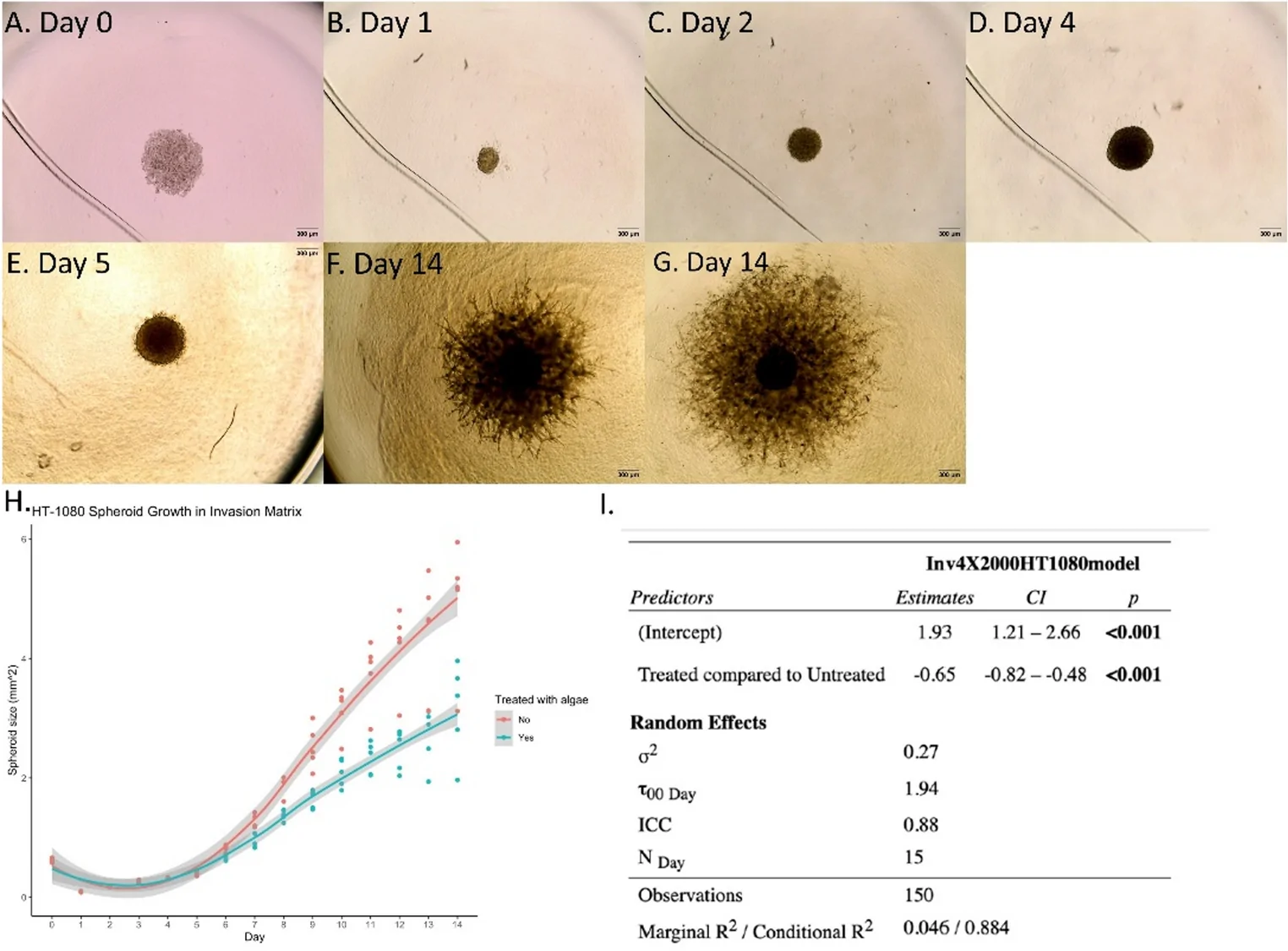
*Ulva Lactuca* Extract Treatment Reduced HT-1080 Spheroid Invasiveness. On Day 0, 2,000 HT-1080 cells were aggregated by centrifugation (**A**). Each spheroid was photographed under a microscope and spheroid area was monitored daily for 2 weeks (*n* = 5 for each group). The compacted spheroid increased in size over time between Day 1 and Day 4 (**B**–**D**). On Day 4, invasion matrix was added to all wells, and HT-1080 spheroids were treated with and without aqueous *U. lactuca* algal extract. Solid lines represent the best-fit line for spheroid size over time calculated using LOESS. The gray shaded regions indicate the 95% confidence intervals (**H**). Statistical analysis using a linear mixed-effects model confirmed a significant reduction in the invaded area of the treated group compared to the control (*p* < 0.001; I). The model estimated a significant fixed effect of treatment (−0.65 mm^2^; 95% CI: −0.82 to −0.48), indicating a consistent reduction in integrated invasive burden between Day 3 to Day 14

Following the observation of reduced spheroid sizes across three different cancer cell lines, subsequent studies investigated whether *U. lactuca* aqueous extract also inhibit spheroid invasion. Initial 3D invasion assays were conducted using HT-1080, HT-29 and LNCap cells to assess invasive phenotypes. Our results showed that only spheroids formed by HT-1080 cells produced radial invasion patterns (Fig. 4F–G) under the tumor microenvironment tested [30]. Therefore, the effects of *U. lactuca* extract on invasion were evaluated using HT-1080 cells.

Each HT-1080 fibrosarcoma spheroid was formed from 2000 cells in a ULA well of a 96-well plate and monitored for 15 days for spheroid growth and invasive phenotype. After a spheroid compaction phase (Fig. 4A—B), the size of each spheroid expanded over time between Day 2 (Fig. 4C) and Day 14. On Day 4, invasion matrix (IM) was added to all wells to simulate ECM, and aqueous extract of *U. lactuca* was added to those in the treated group (Fig. 4D). By Day 5 (Fig. 4E), the emergence of cell membrane protrusions (invadopodia/filopodia) indicated active ECM degradation and subsequent radial migration away from the spheroid core. Spheroid invasiveness was assessed by measuring the area occupied by the invaded HT-1080 spheroid. LOESS regression revealed a significant and progressive divergence in the total invaded area starting on Day 7, with the most pronounced separation occurring between Day 8 and Day 14 (Fig. 4F–G). Treatment with *U. lactuca* extract suppressed HT-1080 invasion expansion by 42.5%, compared to the untreated control between Day 4 and Day 14 (2.7 mm^2^ vs. 4.7 mm^2^; Fig. 4H). This inhibition was highly robust (*p <* 0.001; Fig. 4I), with 95% confidence intervals showing complete isolation from Day 8 through the study’s end. The widening gap between these cohorts underscores the extract’s ability to interfere with the sustained migratory signaling required for mesenchymal invasion.

To evaluate the efficacy of *U. lactuca* extract in a spatially relevant context, changes in spheroid radius were compared relative to the Day 4 baseline (*r* = 0.308 mm) in both cohorts. While untreated control spheroids reached a 3-fold increase in radius (0.977 mm) by Day 10, the *U. lactuca*-treated group exhibited a 4-day delay, reaching this milestone only at the study’s conclusion on Day 14. Suppression of radial infiltration is particularly consequential in 3D systems as the volume of a sphere scales with the cube of the radius ($\:V=\frac{4}{3}\pi\:{r}^{3}$); thus a 3-fold increase in radius corresponds to a 27-fold increase in spherical tumor volume. By delaying radial expansion, *U. lactuca* treatment substantially reduced the integrated invasive burden, as quantified by AUC, reflecting sustained inhibition of invasive outgrowth over the experimental timeframe.

In summary, the longitudinal analysis of 3D multicellular tumor spheroids confirms that aqueous *U. lactuca* extract exerts a potent, multi-lineage inhibitory effect on both tumor expansion and mesenchymal invasion. By suppressing growth across fibrosarcoma (HT-1080), colorectal (HT-29), and prostate (LNCaP) lines (overall 20%*–*26% reduction in spheroid size), the extract demonstrated a broad-spectrum capacity to disrupt proliferative kinetics and extend doubling milestones. Most notably, the 42.5% reduction in the invasive area of HT-1080 spheroids—and the corresponding four-day delay in reaching critical radial expansion thresholds—suggests potential to interfere with processes associated with metastatic progression. By significantly reducing the integrated invasive burden, shown by AUC, and preventing a potential 27-fold increase in volumetric expansion, *U. lactuca* effectively constrains the ‘invasive reach’ of aggressive malignancies. These findings provide evidence that *Ulva*-derived compounds can modulate the growth trajectories and spatial dissemination of human cancer cells in a physiologically relevant 3D environment.

## Discussion

In summary, our longitudinal analysis demonstrates that aqueous *U. lactuca* extract exerts inhibitory effects consistent with potential anticancer activity in 3D tumor spheroid models. Compared to prior studies conducted in 2D monolayer systems, our findings show that *U. lactuca* extract suppresses spheroid growth, delays tumor growth kinetics, and reduces cumulative tumor burden, as well as invasive expansion under diffusion-limited conditions. Demonstrating efficacy in 3D models provides a more physiologically relevant evaluation compared to 2D models, as these systems recapitulate key features of solid tumors, including hypoxia, extracellular matrix deposition, and limited drug penetration.

Beyond reductions in spheroid size, the delayed tumor growth kinetics observed in this study have important translational implications, as tumor doubling time is a clinically relevant parameter associated with disease aggressiveness and cumulative tumor burden (measured by AUC). Even modest extensions in growth milestones can substantially limit tumor expansion over time, thereby reducing the integrated malignant exposure experienced throughout the treatment period. This growth delay suggests that *U. lactuca* extract exerts a tumor-modulating effect. As a result, tumor mass can be maintained below potential critical thresholds associated with systemic inflammation and cancer-induced cachexia.

The concurrent inhibition of radial invasion in HT-1080 spheroids further emphasizes the clinical relevance of these findings. Local invasion and extracellular matrix remodeling are key determinants of intravasation and metastatic progression, particularly in mesenchymal malignancies such as fibrosarcoma. Because a spherical tumor volume scales with the cube of the radius, relatively small delays in invasive outgrowth can translate into disproportionately large reductions in overall tumor burden; in this study, preventing a three-fold increase in spheroid radius effectively averted a 27-fold increase in volumetric expansion. Collectively, these findings suggest that *U. lactuca* extract may have potential as a complementary therapeutic strategy, pending further validation. By slowing proliferation, restricting invasion, and preserving a critical therapeutic window during which immune surveillance or combination therapies may more effectively target a weakened tumor mass.

While the current study successfully demonstrates the antiproliferative potential of *U. lactuca* extract against HT-29, HT-1080, and LNCaP spheroids, several limitations must be considered. First, the findings are based on in vitro systems and do not fully capture the complexity of in vivo tumor biology, including immune interactions, vascularization, and systemic metabolism. Furthermore, this research utilized a fixed-dose screening approach (6.25 mg/mL of crude aqueous extract, corresponding to an estimated 0.56–2.25 mg/mL ulvan) to establish a baseline of bioactivity in a complex 3D architecture. While the reduction in spheroid volume at this concentration is statistically significant, the absence of a complete dose-response curve limits the ability to define the precise IC_50_ value within the 3D microenvironment. Furthermore, the high concentration required for effect in 3D—likely due to the mass-transfer resistance and the dense extracellular matrix of the spheroid—highlights the need for future studies to focus on three key areas: (1) Fractionation: identifying whether the observed effect is driven by the sulfated polysaccharide ulvan or other bioactive compounds, (2) Selectivity: evaluating the extract’s toxicity against non-cancerous 3D models to determine the therapeutic window at this dosage, and (3) Mechanistic profiling: utilizing transcriptomic and proteomic analyses to identify the molecular pathways that modulate these anti-neoplastic effects. Given prior reports that ulvan-rich fractions can modulate ECM remodeling enzymes, including matrix metalloproteinases [32, 33], future studies will investigate whether altered protease–inhibitor balance contributes to the observed reduction in spheroid invasion. Despite these limitations, this study provides crucial proof of concept for the use of marine-derived extracts in treating various cancer models in a physiologically relevant 3D context.

## Conclusions

Using 3D tumor spheroids models, this study demonstrates that *U. lactuca* aqueous extract inhibits tumor spheroid expansion across fibrosarcoma, colorectal, and prostate cancers, and reduces invasive behavior in the fibrosarcoma. The observed delays in growth kinetics and suppression of ECM-driven invasion indicate that the extract can modulate key processes associated with tumor progression in vitro.

While these findings highlight the potential of marine-derived compounds as candidates for anticancer development, further studies are required to identify the active components, define dose-response relationships, and validate these effects in in vivo systems.

Overall, this work supports the use of 3D tumor spheroid models as a robust platform for evaluating marine natural products and provides a foundation for future mechanistic and translational investigations of *U. lactuca* bioactives.

## Data Availability

No datasets were generated or analysed during the current study.

## Abbreviations

3D: Three dimensional
AUC: Area-under-the-curve
LOESS: Locally estimated scatterplot smoothing
IARC: International Agency for Research on Cancer
WHO: World Health Organization
IC50: Half-maximal inhibitory concentration
DMBA: 7,12-dimethylbenz[a]anthracene
ECM: Extracellular matrix
CCA: Culture Collection of Algae
ULA: Ultra-low attachment
